# Placement-Dependent Accuracy of a Smartphone-Based Sensor Application Compared to an Accelerometer-Based System for Measuring Physical Activity in Healthy Adults: A Validation Study

**DOI:** 10.3390/s26072033

**Published:** 2026-03-25

**Authors:** Mette Garval, Louise Pedersen, Lars M. Pedersen, Ane Kathrine W. d. J. Nielsen, David H. Christiansen, Jeppe Lange, Stefan Wagner

**Affiliations:** 1 Elective Surgery Center, University Clinic for Interdisciplinary Orthopaedic Pathways, Regional Hospital Central Jutland, Falkevej 1, 8600 Silkeborg, Denmark; 2 Department of Clinical Medicine, Aarhus University, Vennelyst Boulevard 4, 8000 Aarhus, Denmark; 3 Department of Electrical and Computer Engineering, Aarhus University, Finlandsgade 22, 8200 Aarhus, Denmark; 4 Department of Orthopaedic Surgery, Regional Hospital Horsens, Sundvej 30, 8700 Horsens, Denmark

**Keywords:** validation study, measurement accuracy, activity recognition, smartphone-based sensors, wearable sensors, accelerometry

## Abstract

**Highlights:**

**What are the main findings?**

- Activity recognition classification accuracy of the BeSAFE+ smartphone sensor application varied by phone placement and activity type.
- Cycling was most accurately detected with the phone in the front pocket using the Sampling Application Programming Interface (API), and non-cycling activities were most accurately classified with the phone in the back pocket using the delay-compensated Transition API.

**What are the implications of the main finding?**

- Smartphones may serve as a feasible, low-cost alternative to wearable activity trackers in rehabilitation practice, if placement and API selection are tailored to the target activity.
- These findings are particularly relevant for research conducted in resource-limited settings and for studies requiring long-term follow-up.

**Abstract:**

Accurately monitoring physical activity, including stationary cycling on an exercise bike, is important in managing chronic diseases and rehabilitation after lower limb surgery. This study aimed to validate a new smartphone-based sensor application (the BeSAFE+) for activity recognition and step counting across five phone placements, using the SENS Motion^®^ system as a reference standard, and observed activity time as ground truth. In a laboratory-based study, 20 participants performed walking, brisk walking, running, high- and low-intensity cycling, sitting, standing, and lying activities while carrying five smartphones placed in the front and back trouser pockets, a backpack, a running armband, and a fanny pack, and wearing the activity tracker. The front pocket placement had the most accurate classification during cycling activities (89–93%) versus SENS Motion^®^ (96–98%). For other activities, the highest overall classification accuracy was achieved with the phone in the back pocket. Overall, the SENS Motion^®^ activity tracker demonstrated higher classification accuracy than most smartphone placements across all activities, except for running. Nevertheless, several smartphone placements and Application Programming Interface (API) approaches achieved activity recognition and step count estimates that were not significantly different from the SENS Motion^®^ activity tracker, indicating that smartphone-based activity recognition can be valid under specific conditions.

## 1. Introduction

Regular physical activity (PA), defined as “any bodily movement produced by skeletal muscles that results in energy expenditure” [1], is essential for managing chronic cardiometabolic and musculoskeletal diseases, obesity, and certain cancers [2,3]. Accurate PA monitoring is important for evaluating adherence, dose–response relationships, and rehabilitation outcomes.

Traditionally, self-report methods such as diaries and interviews have been widely used due to their low cost and simplicity. However, these approaches are prone to recall bias and subjectivity, with users often overestimating activity levels, especially over longer recall periods [4,5]. More objective methods include indirect calorimetry, direct observation, or physiological measurements, which are more accurate but are costly, time-consuming, and challenging to implement in large-scale or long-term studies [5,6]. Advances in Human Activity Recognition (HAR) have introduced wearable sensors that enable continuous and objective monitoring of PA [7,8]. While these devices provide valid measurements, they rely on users to upload data, require frequent charging, and may cause skin irritation [9]. Smartphones, which are widely available and typically include accelerometers and gyroscopes, offer a practical, low-cost tool for PA monitoring [10,11].

Existing studies show that smartphones can classify common activities such as walking, running, and standing [12], with performance varying widely (62.6% to 98.7%) depending on the task, model, and smartphone placement [13]. Smartphone-based HAR systems, however, are influenced by the device’s placement on the body [12], with more stable placements such as trouser pockets generally yielding higher accuracy [14]. In contrast, less stable placements, like in a backpack or on the arm, tend to result in lower recognition accuracy [14,15]. Despite technical improvements, only a limited number of studies have validated smartphone-based HAR against body-worn motion sensors. Hekler et al. compared the accuracy between three different Android smartphones, worn simultaneously on the hip or in the pocket, to an ActiGraph accelerometer worn on the opposite hip for measuring physical activities in 15 participants. The results indicated that smartphones correctly classified sitting, walking and jogging with an accuracy ranging from 78% to 91%, depending on the data processing method used [16]. However, the study found poor performance in classifying activities like cycling and standing, which were excluded from the final analysis. Notably, while smartphone placement (on the hip vs. in the pocket) did not significantly affect the classification accuracy for sitting, walking, and jogging, the findings still underscore the need for further investigation into the effect of other smartphone placements [16]. Another study by Minh et al. using a large dataset found that smartwatch accelerometers outperformed smartphones worn in the pocket in classifying activities such as walking, jogging, and stair climbing, likely due to their more stable wrist placement [17]. However, these studies focus on single placement conditions, which limit the generalizability of their findings. Therefore, the placement-dependent accuracy of smartphones requires further evaluation, particularly in comparison to body-worn sensors, to ensure reliable monitoring of physical activity and, importantly, the accurate differentiation of cycling from other activities. This may lead to preferred phone placements for recognizing individual activities.

Given the broad range of activities involved in rehabilitation, it is essential to assess multiple types of physical activities, such as standing, walking, running and cycling. Stationary cycling is a cornerstone physiotherapy intervention, widely used in rehabilitation for patients with chronic cardiac or pulmonary conditions, as well as those with hip or knee osteoarthritis or recovering from lower limb surgery [18,19,20,21]. However, its accurate detection using smartphone-based systems remains underexplored. The present study therefore aimed to validate a newly developed smartphone-based sensor application for activity recognition and step counting across five phone placements (front and back trouser pockets, backpack, armband, and fanny pack) using the SENS Motion^®^ system as a reference standard, and observed activity time as ground truth. The validation covered multiple activities, with particular emphasis on stationary cycling, given its clinical importance in lower-limb osteoarthritis rehabilitation and postoperative recovery. We hypothesized that classification accuracy would vary by phone placements, with more stable positions expected to achieve higher classification accuracy than the armband. By systematically evaluating placement-dependent classification accuracy across activities, this study offers novel methodological insights into the practical use of smartphone-based monitoring for rehabilitation, with additional relevance for research in resource-limited settings and long-term follow-up studies.

## 2. Materials and Methods

This study was designed as a laboratory-based validation study with volunteer participation. The participants conducted a range of activities (walking, brisk walking, running, cycling, sitting, standing, lying) in a laboratory setting at Aarhus University, Denmark. While conducting the activities, the participants had five separate smartphones, placed in different positions, and an activity tracker placed on the left thigh, all collecting data. A research facilitator recorded the observed start and stop time of an activity in a web-application designed for the purpose “https://data-collection-app-three.vercel.app/ (accessed on 10 April 2025)”. Observed start and stop times were used as ground truth to align and evaluate predictions.

### 2.1. Study Population and Recruitment

Participants were recruited voluntarily from the Department of Electrical & Computer Engineering at Aarhus University, Denmark, between April and May 2025. The sample, therefore, comprised students and faculty members. Inclusion criteria were age ≥18 years, ability to read and understand Danish, and absence of injuries or physical limitations interfering with normal treadmill or exercise bike use. No financial or other compensation was provided. Basic demographic characteristics (age, sex, weight, height) were collected for all participants. All participants provided written informed consent, and data were collected and analyzed anonymously. The study was approved by Aarhus University’s Research Ethics Committee.

Participants were encouraged to wear their own trousers to increase real-world relevance and reflect typical smartphone-carrying conditions. If participants’ trousers lacked both a front and a back pocket, the project staff provided training trousers.

No formal sample size calculation was performed, as the study was designed as a methodological validation study.

### 2.2. Experimental Design

The study used a treadmill (Kilberry PMT-4550, Kilberry, Shanghai, China), an exercise bike (Concept2 BikeErg PM5, Concept2 Inc., Morrisville, VT, USA), a chair without armrests, a bed, a backpack, a fanny pack of sufficient size to contain a smartphone, and a running armband suitable for smartphone carriage. Five Android smartphones of identical make and model (Samsung Galaxy A53 5G, Samsung Electronics Co., Ltd., Suwon, Republic of Korea) running Android 14 with the application (app) BeSAFE+ installed were attached to the participant.

The BeSAFE+ app was developed by the authors (Department of Electrical and Computer Engineering, Aarhus University, Aarhus, Denmark) [22] using Microsoft .NET 8.0 MAUI (.NET Multi-platform App UI: “https://dotnet.microsoft.com/en-us/apps/maui (accessed on 10 April 2025)”, enabling cross-platform deployment across Android, iOS, and Tizen-based devices. However, the present study was limited to the Android platform due to differences in available features between device models and operating systems (MMotionActivityManager, Apple Developer Documentation: “https://developer.apple.com/documentation/coremotion/cmmotionactivitymanager (accessed on 10 April 2025)”. The app continuously monitored activity in the background, storing data locally before periodically summarizing and transmitting data to a secure Research Electronic Data Capture (REDCap) database server [23], located within the secure server facility at the Aarhus University, Denmark. Activity data was obtained through the Android Activity Recognition Application Programming Interface (API), including both Activity Recognition Transition API (transition API) and Activity Recognition Sampling API (sampling API) “ActivityRecognitionClient, Google Play services: https://developers.google.com/android/reference/com/google/android/gms/location/ActivityRecognitionClient (accessed on 10 April 2025)”.

**Figure 1:**
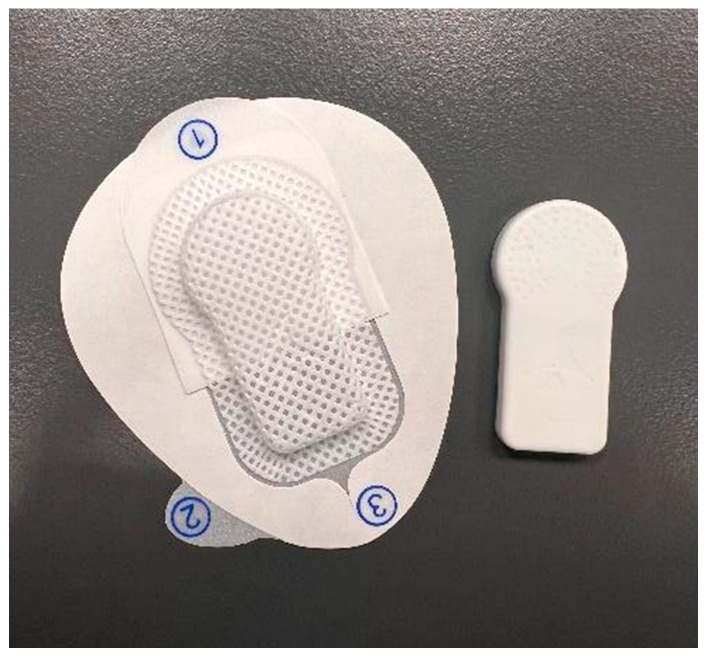
SENS Motion^®^ body sensor and adhesive patch. Numbers (1–3) indicate the stepwise sequence for applying the adhesive patch to attach the sensor.

As a reference standard, physical activity was recorded simultaneously using the SENS Motion^®^ accelerometer system (SENS Innovation ApS, Copenhagen, Denmark). The system includes a wearable wireless sensor (Figure 1), a smartphone app for data transfer, and a web-based platform for visualization and extraction. The sensor was attached to the front mid-thigh of the participant’s left leg, following the developer’s guidelines, using an adhesive patch, after cleaning the skin with isopropyl alcohol wipes. The SENS Motion^®^ system was chosen for its thigh-mounted placement, allowing direct measurement of lower-limb movements and suitability for activities with minimal upper-body motion, such as stationary cycling, which was a key focus of this study. Raw triaxial acceleration data were processed with the Acti4 algorithm (National Research Centre for the Working Environment, Copenhagen, Denmark). This algorithm classifies activity into ten predefined categories (lying, sitting, standing, standing with movement, walking, stair ambulation, running, cycling, rowing, and non-wear) [24]. The SENS Motion^®^ system has shown valid and reliable assessment of physical activity and sedentary behavior in adults and children, though its step detection is less accurate at very slow walking speeds [25,26,27]. The Acti4 algorithm was selected as the reference classification method due to its established ability to identify cycling activity from raw acceleration patterns, which was a primary focus of the present study. The use of this algorithm is in line with the manufacturer’s recommendations for the SENS Motion^®^ system to ensure high precision in classifying dynamic activities.

**Figure 2:**
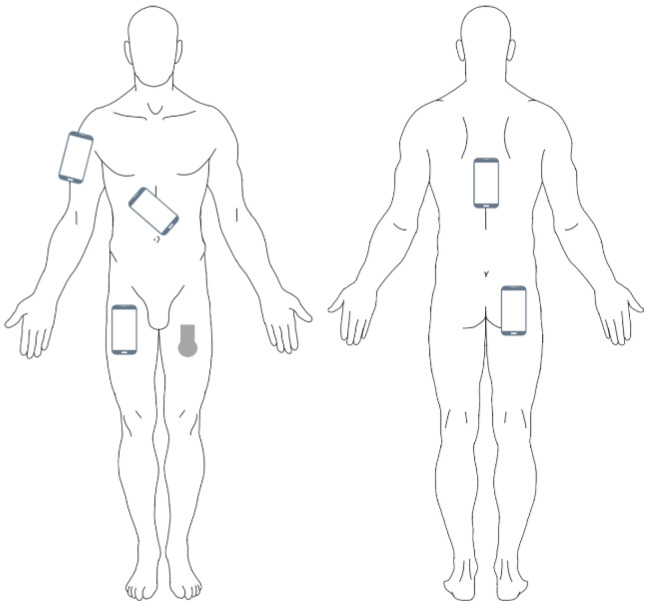
Placement of the smartphones and the SENS Motion^®^ activity tracker.

Participants performed the activities listed in Table 1 in the predefined sequence, while simultaneously carrying all five smartphones in different placements, along with the SENS Motion^®^ activity tracker. Each activity was performed for a predefined minimum duration (as specified in Table 1), ensuring a balanced dataset for the subsequent classification performance evaluation. The evaluated smartphone placements were: right front trouser pocket, right back trouser pocket, backpack worn on the back, running armband worn on the right upper arm, and a fanny pack worn across the body (Figure 2). During the “lying still” activity, the backpack was placed on the floor for all participants. If a smartphone could not be worn in its designated placement for a given activity due to clothing obstacles (e.g., lack of suitable pockets), it was placed on a table and excluded from subsequent analyses for that exact smartphone placement.

### 2.3. Data Processing

Data from smartphones and the SENS Motion^®^ activity tracker were extracted after all experimental sessions were completed. Smartphone data include triaxial accelerometer and gyroscope signals, step counts, and activity classifications from the Android Activity Recognition APIs. Local SQLite databases were retrieved from each device and exported to comma-separated value (.CSV) files for subsequent processing. Activity classification data from smartphones were processed to calculate which values were highest and correctly classified at each time points. Smartphone predictions were then processed to determine classification accuracy at each time point relative to the observed activity labels. For the collected Sampling API predictions, an activity was considered correctly classified when the value with the highest confidence value corresponded to the observed activity. For the Transition API, classifications were considered correct when the most recently detected transition matched the observed activity. Short periods classified as “Unknown” were handled with a last observation carried forward (LOCF) approach. In this procedure, “Unknown” labels were removed and replaced with the most recently identified activity to maintain temporal continuity in the activity classification. Analyses were performed with and without application of the LOCF filter.

For the Transition API, both raw transition outputs and delay-compensated transitions were analyzed. The mean detection delay was calculated from the recorded transition data. Delay-compensated transitions were generated by subtracting the average delay from detected transition times. This improved temporal alignment with observed activities. If the Transition API misclassified the activity, that data was excluded from the calculation of mean detection delay. In addition, the activity categories “Tilting” and “InVehicle”, which are inherent outputs of the applied smartphone API, were retained in the analysis. Although these activities were not explicitly performed by the participants, they were included to capture potential misclassifications of performed activities.

Data from the SENS Motion^®^ activity tracker was retrieved from the secure SENS cloud. The SENS platform offers multiple activity classification algorithms. For this study, the Acti4 algorithm was selected beforehand. Activities were labeled using the categories from Table 2. Based on the observed start and end times, the duration in seconds was calculated for each activity category.

Step count data from smartphones were recorded on a per-minute basis and compared with step counts from the SENS Motion^®^ activity tracker. After synchronization, raw accelerometer and gyroscope data from the smartphones were deleted to reduce storage requirements. Step counts per activity were estimated using two interpolation approaches: (1) a uniform distribution method, assuming steps were evenly distributed within each recorded minute, and (2) an average of fully contained steps method, including only minutes fully contained within a single activity. The latter approach was applied only to activities lasting two minutes or longer. For all data sources, classification performance was quantified as the percentage of correctly predicted seconds relative to the observed ground truth.

### 2.4. Statistical Analysis

Data were analyzed using Python 3.12.7 and MATLAB R2023a. Normality of continuous variables was assessed using Q–Q plots and the Anderson–Darling test. As all variables were non-normally distributed, non-parametric analyses were applied, with differences between groups assessed using the Kruskal–Wallis test with a degree of freedom of five. A significance threshold of *p* < 0.05 was applied. The agreement between the SENS Motion^®^ activity tracker and observed ground truth was evaluated using a confusion matrix. Classification accuracy was reported separately for each Android API. For the Sampling API, data were analyzed both with and without the LOCF filter for each phone placement, enabling comparison of classification performance before and after handling “Unknown” values. For the Transition API, both raw and delay-compensated transitions for each phone placement were analyzed, allowing comparison of activity-detection classification accuracy before and after each data adjustment. For each participant, the number of correctly and incorrectly classified seconds was calculated and summarized in combined confusion matrices for each activity category.

The statistical analysis also included an analysis of the pedometer behavior during the observed activities. Step counts from smartphones were compared with step counts from the SENS Motion^®^ activity tracker. Differences between activity categories and step counts within the start and end times of each activity were evaluated using the Kruskal–Wallis test, followed by post hoc analysis using multiple comparisons plots with the Tukey–Kramer procedure to control the Type I error rate] [28]. Step counts were derived using two interpolation approaches: uniform distribution across the activity duration and averaging of fully contained steps within complete minutes. Both approaches were applied in the comparison plots. For activities lasting less than two minutes, step counts were recorded as zero steps per minute if no fully contained minutes were available.

## 3. Results

A total of 20 participants between the ages of 20 and 52 years old, including 3 females and 17 males, were included. The distributions of age, sex, height, and weight are presented in Table 3. Detailed participant characteristics and excluded data for all 20 participants are provided in Appendix A, Table A1.

Three participants did not have a suitable back pocket and data from that placement was excluded for those participants.

### 3.1. SENS Motion^®^ Data

**Figure 3:**
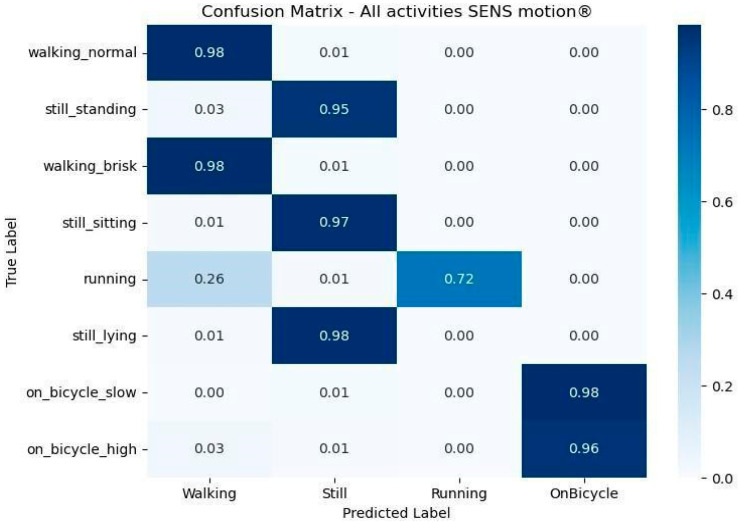
Confusion matrix shows the observed versus predicted activity from the SENS Motion^®^ activity tracker as reference standard. The *x*-axis represents the activity categories predicted by the reference standard. The *y*-axis represents the true observed activity. Darker blue indicates a higher percentage of predictions within the category.

Figure 3 presents the confusion matrix based on data from the SENS Motion^®^ activity tracker, using the category mappings defined in Table 2. Overall, the matrix indicates a strong agreement between the SENS Motion^®^ activity tracker and the observed ground truth across most activities. Cycling activities were recognized with high classification accuracy, with low-intensity cycling at 98% and high-intensity cycling at 96%. Normal and brisk walking were classified with 98% accuracy, and still activities (standing, sitting, lying) with 95–99%. Running was more frequently misclassified, with 72% correctly identified and 26% mislabeled as walking. Note that the “Unknown” category is not present since no “Unknown” activity was detected by the SENS Motion^®^ activity tracker during the observed activities.

### 3.2. Smartphone Data

**Figure A1:**
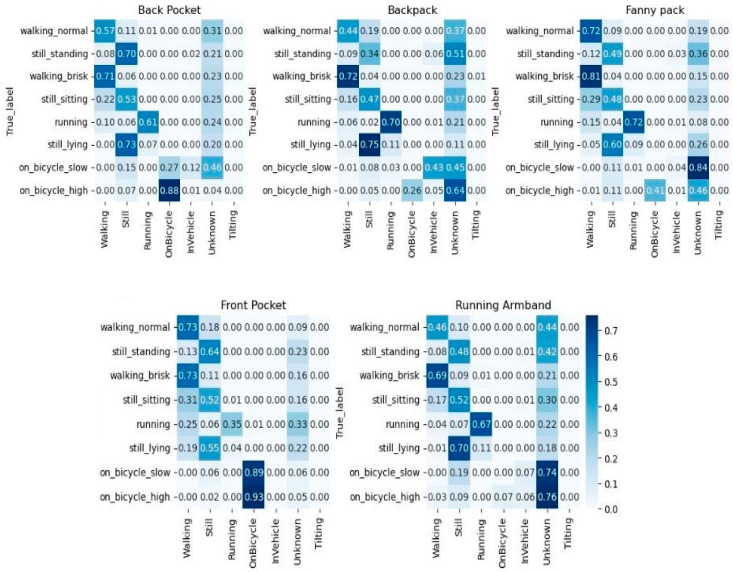
Confusion matrices showing the observed versus predicted activity from the Sampling API. The *x*-axis represents the activity categories predicted by the smartphone using the Sampling API. The *y*-axis represents the true observed activity. Darker blue indicates a higher percentage of predictions within the category.

The Sampling API demonstrated activity-dependent performance, with the highest classification accuracies observed during brisk walking (≥69% across all placements). Classification performance varied across phone placements. Average agreement across all activities was highest for the front pocket (66.8%), followed by the back pocket (62.5%), the fanny pack (52.9%), the running armband (44.9%), and the backpack (41.9%). For cycling activities, agreement was highest when the smartphone was placed in the front pocket, reaching 89% for low-intensity and 93% for high-intensity cycling. The back pocket placement reached 88% agreement for high-intensity cycling but showed lower agreement for low-intensity cycling (27%). Detailed confusion matrices are provided in the Appendix A, Figure A1.

**Figure 4:**
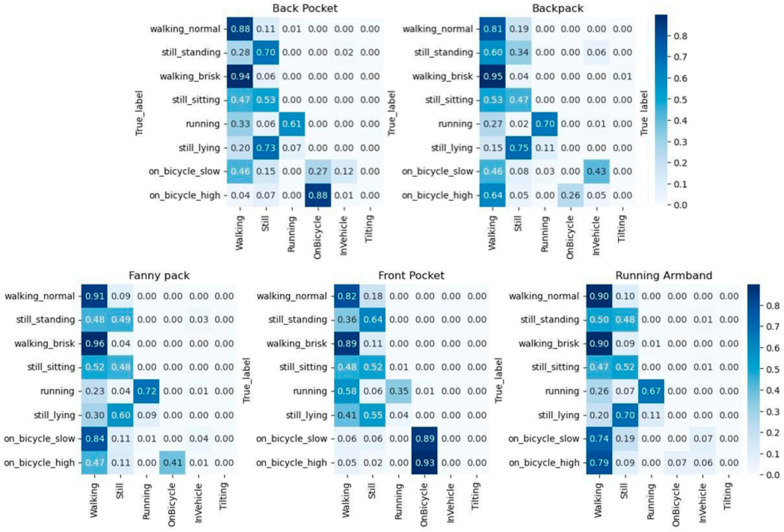
Confusion matrices showing the observed versus predicted categories from the Sampling API for each phone placement. The *x*-axis represents the activity categories predicted by the smartphone using the Sampling API. The *y*-axis represents the true observed activity. Darker blue indicates a higher percentage of predictions within the category.

Using the LOCF filter to remove values, where the “Unknown” category had the highest value increased agreement across all placements, again with the highest classification accuracies observed during brisk walking (≥89% across all placements) as seen in Figure 4. Average agreement across all activities was 69.9% for the front pocket, 69.3% for the back pocket, 57.1% for the fanny pack, 53% for the running armband, and 50.4% for the backpack.

**Figure A2:**
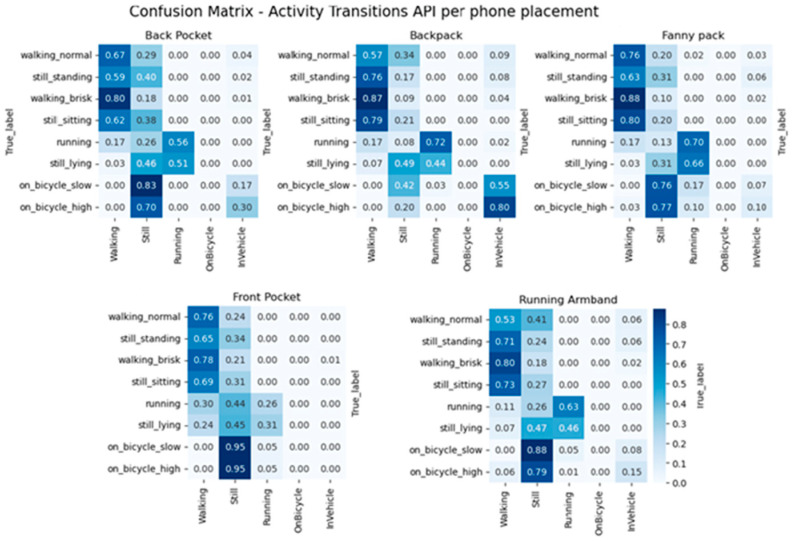
Confusion matrices showing the observed versus predicted activity from the Transition API. The *x*-axis represents the activity categories predicted by the smartphone using the Transition API. The *y*-axis represents the true observed activity. Darker blue indicates a higher percentage of predictions within the category.

The confusion matrices for the raw Transition API yielded varying results in predicting activities, ranging from 17% to 88% (Appendix A, Figure A2). The average classification accuracy across all activities was 40.9% for the back pocket, 39.5% for the fanny pack, 36.8% for the running armband, 36.3% for the backpack, and 36.3% for the front pocket.

**Figure 5:**
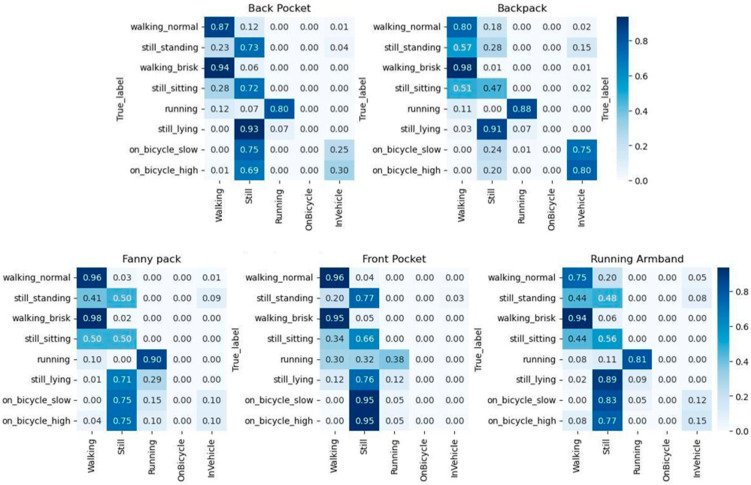
Confusion matrices showing the observed versus predicted categories from the delay-compensated Transition API for each phone placement. The *x*-axis represents the activity categories predicted by the smartphone using the delay-compensated Transition API. The *y*-axis represents the true observed activity. Darker blue indicates a higher percentage of predictions within the category.

For the Transition API, recorded transitions were adjusted by the calculated average detection delay to account for the time lag in correctly identifying activities. This detection delay was calculated to be 37.64 s (standard deviation: 19.06) across all placements and activities. This delay compensation improved agreement with the observed activities shown in Figure 5. Average classification accuracy across all activities for each phone placement was 62.4% for the back pocket, 56.9% for the fanny pack, 56% for the front pocket, 55.4% for the running armband, and 48.7% for the backpack.

### 3.3. Sampling API and Transition API Versus SENS Motion^®^

Statistically significant differences in classification accuracy were observed between the three API versions (Sampling API with LOCF filter, raw Transition API, and delay-compensated Transition API) across all activity categories and individual activities (*p* < 0.05 for all comparisons) (Appendix A, Table A2 and Table A3).

**Figure 6:**
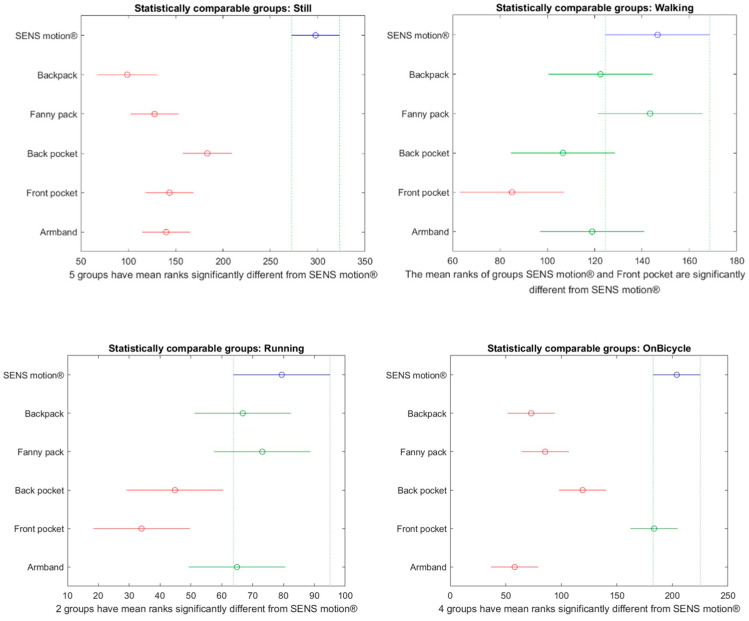
Multiple Comparisons Plots based on the SENS Motion^®^ activity tracker and the Sampling API using LOCF filter data. The *x*-axis represents the mean ranks used in the non-parametric pairwise comparisons. The *y*-axis provides the phone placements and the SENS Motion^®^ activity tracker (visualized in blue). Phone placements highlighted in red differed significantly from the SENS Motion^®^ activity tracker, while green color indicates no significant difference.

**Figure 7:**
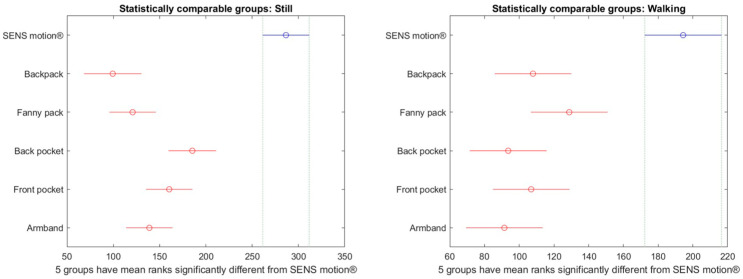

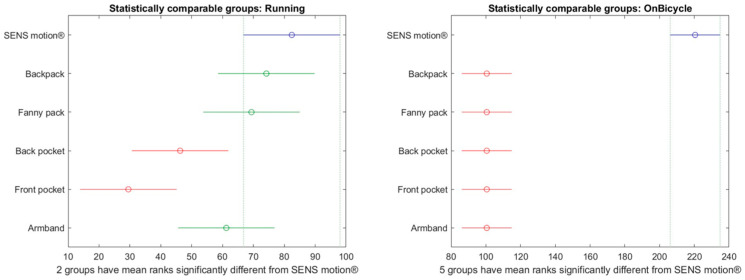
Multiple Comparisons Plots for the SENS Motion^®^ activity tracker and Transition API (raw) data. The *x*-axis represents the mean ranks used in the non-parametric pairwise comparisons. The *y*-axis provides the phone placement and the SENS Motion^®^ activity tracker (visualized in blue). Phone placements highlighted in red differed significantly from the SENS Motion^®^ activity tracker, while green color indicates no significant difference.

**Figure 8:**
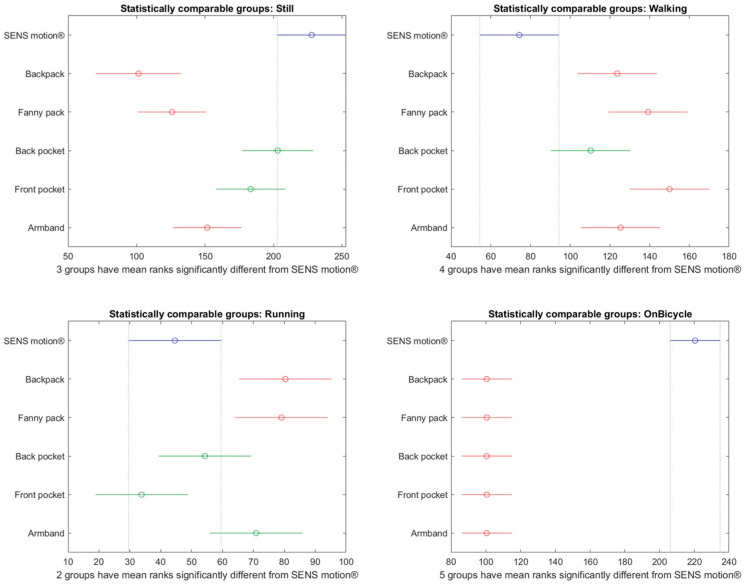
Multiple comparison plots based on the SENS Motion^®^ activity tracker and the delay-compensated Transition API data are shown. The *x*-axis represents the mean ranks used in the non-parametric pairwise comparisons. The *y*-axis provides the phone placement and the SENS Motion^®^ (visualized in blue). Phone placements highlighted in red differed significantly from the SENS Motion^®^ activity tracker, while green color indicates no significant difference.

**Figure A3:**
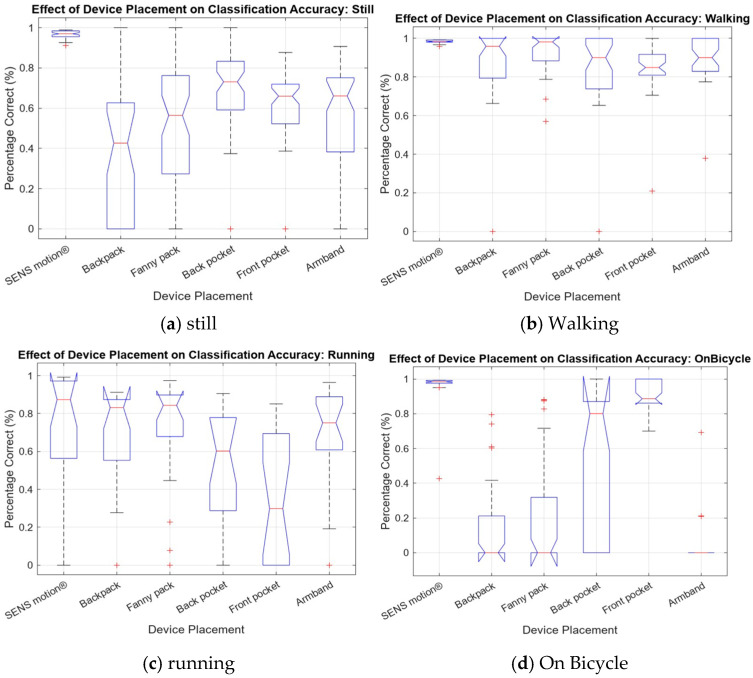
Notched boxplots of data from the Sampling API grouped by activity.

**Figure A4:**
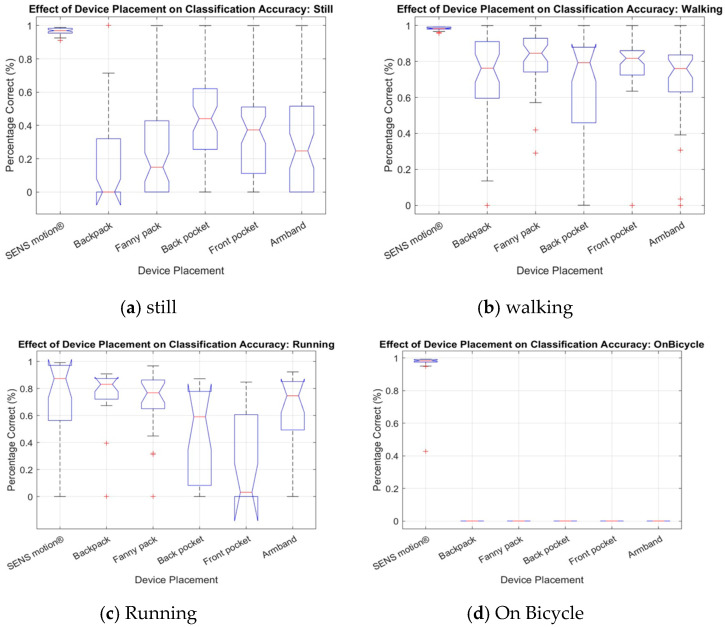
Notched boxplots of data from the Transition API (raw) grouped by activity.

**Figure A5:**
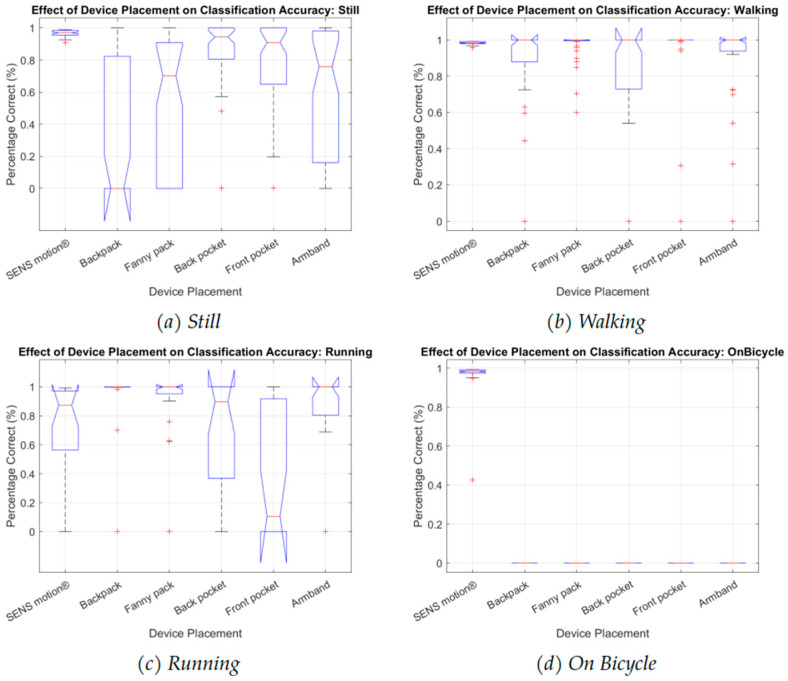
Notched boxplots of data from the Transition API (compensated) grouped by activity.

Figure 6, Figure 7 and Figure 8 present multiple comparisons highlighting groups that differed significantly from the SENS Motion^®^ activity tracker for each activity. Figure 6 shows results based on the Sampling API using the LOCF filter, Figure 7 on the raw Transition API, and Figure 8 on the delay-compensated Transition API. Boxplots comparing classification performance across phone placements and the SENS Motion^®^ tracker for each activity and API version are provided in the Appendix A, Figure A3, Figure A4 and Figure A5.

Compared to the Sampling API with the LOCF filter, all phone placements showed a statistically significant difference from the SENS Motion^®^ activity tracker when standing still, as seen in Figure 6. The most accurate results were observed during walking, where only the front pocket placement showed a statistically significant difference compared with the SENS Motion^®^ activity tracker. For cycling activities, the front pocket placement did not differ significantly from the SENS Motion^®^ activity tracker. The performance was significantly different between the SENS Motion^®^ activity tracker and the raw Transition API data in all activities except running, as seen in Figure 7.

Using the delay-compensated Transition API, neither the back nor the front pocket placement differed significantly from the SENS Motion^®^ activity tracker when participants were still. During walking, only the back pocket placement showed a non-statistically significant difference, as illustrated in Figure 8.

### 3.4. Step Analysis

To identify the step counts recorded by the smartphone during all activities across different phone placements, the SENS Motion^®^ activity tracker was used as a reference standard. Significant differences in step counts were observed between phone placements across all activities, with *p*-values < 0.05 for all comparisons (Appendix A, Table A4).

**Figure 9:**
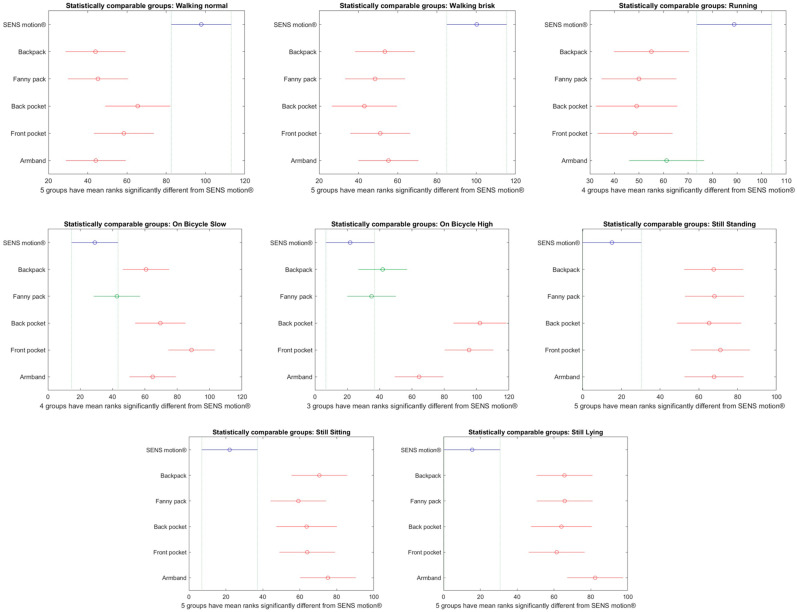
Multiple comparisons plots highlighting significantly different groups from the SENS motion^®^ activity tracker based on activity, using the registered steps per minute. Steps are interpolated using the uniform distribution method. The *x*-axis represents the mean ranks used in the non-parametric pairwise comparisons. The *y*-axis provides the phone placement and the SENS Motion^®^ (visualized in blue). Phone placements highlighted in red are significantly different from SENS motion^®^: those positioned to the left of SENS motion^®^ counted significantly fewer steps, while those to the right counted significantly more steps. Green color indicates no significant difference from SENS motion^®^.

Significant differences in step counts between SENS Motion^®^ and most phone placements across the majority of activities were also observed in the post hoc analysis (Figure 9).

For walking and running activities, all phone placements recorded significantly fewer steps compared to the SENS Motion^®^ activity tracker, except during running when the phone was placed in the armband (non-significant difference). For still activities, all phone placements recorded significantly more steps than the SENS Motion^®^. For cycling activities, all phone placements recorded significantly more steps compared to the SENS Motion^®^ activity tracker, except when the phone was placed in the fanny pack or backpack (only during high-intensity cycling).

**Figure 10:**
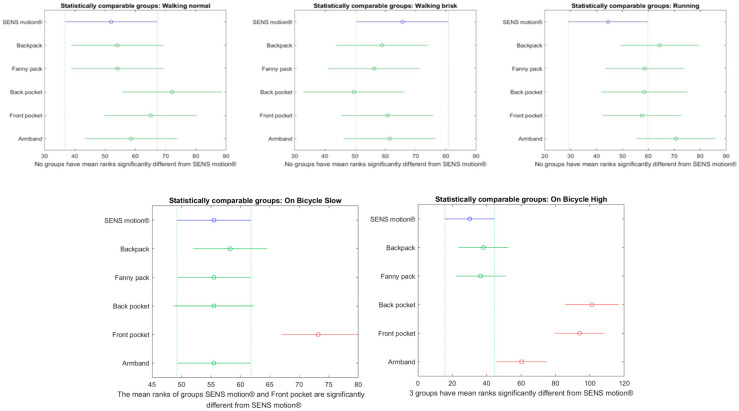
Multiple comparison plots illustrate phone placements that differ significantly from the SENS Motion^®^ activity tracker using the registered steps per minute for each activity. Steps are interpolated using the average of the fully contained steps method. The *x*-axis represents the mean ranks used in the non-parametric pairwise comparisons. The *y*-axis provides the phone placement and the SENS Motion^®^ (visualized in blue). Phone placements highlighted in red differed significantly from the SENS Motion^®^ activity tracker (counted more steps), while green color indicates no significant difference.

A subsequent post hoc analysis was performed to identify which phone placements were most comparable to SENS Motion^®^, and the results are presented in Figure 10. For low-intensity cycling, step counts did not differ significantly between the SENS Motion^®^ activity tracker and the smartphone placement, except when placed in the front pocket. For high-intensity cycling, significant differences were observed for the back pocket, front pocket, and armband placements, while the backpack and fanny pack remained comparable.

## 4. Discussion

### 4.1. Summary of Main Findings

Agreement between the SENS Motion^®^ activity tracker and ground truth was high for all activities (>95%) except running (72%). Smartphone based activity recognition demonstrated similarly high classification accuracy in stationary cycling when the smartphone was placed in the front trouser pocket (89–93%). Across other activities, the highest overall classification accuracy was observed with the phone in the back trouser pocket; however, classification accuracy for each activity varied depending on both the selected API and the smartphone placement. For walking activities, smartphone-based activity recognition showed high agreement with the SENS Motion^®^ activity tracker across most stable placements (75–96%). For running, the highest agreement with the SENS Motion^®^ activity tracker occurred when the smartphone was placed in the backpack or fanny pack (88–90%). In the step-count analysis, based on registered steps per minute, all smartphone placements recorded either higher or lower step counts compared with the SENS Motion^®^ activity tracker. Exceptions occurred for running with the smartphone in an armband and for cycling with the smartphone placed in the backpack or fanny pack, where step counts were comparable.

### 4.2. Performance of Sampling and Transition APIs

The Sampling API demonstrated activity-dependent performance, with the highest classification accuracies observed during brisk walking (≥69% across all placements). Application of the LOCF filter generally improved the Sampling API’s performance, increasing brisk walking classification accuracy to ≥89% across all placements. However, in activities dominated by “Unknown” classifications, such as cycling activity with the phone in the backpack, LOCF can create misleading results by falsely attributing activity to “Walking”. This can potentially be caused by the phone’s position in the backpack, as cycling might cause the phone to slightly jump around in the backpack, changing its orientation more than the SENS Motion^®^ activity tracker being stationarily placed. Hence, the orientation of the phone needs to be considered when using it to detect activity [12].

After applying delay compensation to the raw Transition API data, the classification of still activities improved. For example, standing was correctly classified as a still activity in 77% of the cases for the front pocket placement, although 20% of standing periods were still misclassified as walking. While the delay-compensated Transition API improves the classification accuracy in recognizing transitions to inactivity, it appears less effective in dynamic activities as it failed to classify cycling across all phone placements, even when using delay compensation, which were the primary focus of this study. However, the average delay may vary in other contexts, depending, for example, on smartphone model, manufacturer, and software versions. To minimize such variability in the present study, all smartphones were identical in make and model, and no other applications were active during data collection.

### 4.3. BeSAFE+ Compared to SENS Motion^®^

The SENS Motion^®^ activity tracker outperformed all smartphone placements across most activities. This is consistent with previous validation studies of SENS Motion^®^ conducted in both healthy and hospitalized adult populations [25,26,27]. When comparing the Sampling API (with LOCF applied) to the SENS Motion^®^ activity tracker, the highest agreement was observed during walking. Only the front pocket placement differed significantly from SENS Motion^®^. Across activities, smartphone-based activity recognition consistently performed best during walking, irrespective of phone placement. Similar findings have been reported by Barua et al., who identified walking as the most accurately classified activity across three different phone placements [14]. However, Yuan et al. and Chen et al. underline how body movements are differentiated between individuals, hence the activity performance will differ in connection with step frequency among others [12,13]. This might explain the misclassifications of walking during running, across all phone positions, and the SENS Motion ^®^ activity tracker. Not only can body movement differentiate the comparative results, but using the Transition API instead of the Sampling API also changes the results when compared to SENS Motion^®^.

Using the raw Transition API, agreement with SENS Motion^®^ was limited and primarily observed while running, with the backpack, fanny pack, and armband yielding the most reliable results. Similarly, Barua et al. obtained their highest performance during running using the backpack position [14]. However, as our results show, time lags interfere with performance. Compensating for this delay improved overall activity recognition.

Figure 5 shows that all positions except the front pocket achieved above 80% correct classification. Notably, the front pocket only achieved 72% correct classification despite being one of the phone placements most comparable to the SENS Motion^®^ activity tracker, according to Figure 10. Additionally, the delay-compensating Transition API achieved, for the fanny pack, a classification accuracy of 96% for normal walking and 98% for brisk walking. For the front pocket, it was 96% and 95%, respectively. In comparison, the SENS Motion^®^ activity tracker achieved 98% classification accuracy for both normal and brisk walking. The backpack and fanny pack placements outperformed the SENS motion^®^ activity tracker during running, achieving accuracy scores of 88% and 90%, respectively (Figure 5), This might explain the significant differences in Figure 8, which makes the delay-compensating Transition API a possible alternative in a controlled environment.

Placing the phone in the back pocket and using the Transition API with delay-compensation were overall the most accurate when detecting activities other than cycling. The back pocket yielded an overall classification accuracy of 83% when detecting non-cycling activities, which was non-significantly different from the SENS Motion^®^. In brisk walking, the delay-compensated Transition API achieved classification accuracies between 94% and 98% across phone placements, which were comparable to the 98% classification accuracy obtained by the SENS Motion^®^ activity tracker. Thus, activity recognition performance during brisk walking appeared largely independent of phone placement when using the Transition API with delay compensation.

Cycling activities were most accurately classified by SENS Motion^®^ (96–98%), and by the smartphone placed in the front pocket using the Sampling API with or without with LOCF (89–93%). The high performance of SENS Motion^®^ is likely related to its thigh placement, as the thighs are the primary body part used for this activity. However, the SENS Motion^®^ platform does not provide information on cycling intensity. Estimating cadence-specific intensity (e.g., 40–50 RPM) would require additional processing of raw accelerometer data.

### 4.4. Step Counting Performance

To reduce the storage space requirement, steps were saved in the local application database at a per-minute interval. While this is deemed sufficient for the real-world scenario where changes between activities are not as quick, it reduces the accuracy of the analysis made in this study, as it assumes the steps are uniformly distributed across the time interval, which they were not. Saving the steps in real time as they are collected would solve this issue, however that would quickly increase storage and number of memory-writes in a real-world application.

The consequence of uniformly distributing the measured steps means that some of the steps made during an active activity, such as walking or running, would be carried over into still activities. This means that the steps counted during the active activities are probably underestimated, and the steps in the still activities are overestimated. This was also reflected in the post hoc analysis (Figure 9), where all phone placements in the still activities registered significantly more steps than the SENS Motion^®^ activity tracker and almost all placements in the active activities counted significantly fewer steps. A validation study of SENS Motion^®^ shows that this activity tracker also makes the step counts either above or below the actual number [27]. Using the registered steps, which were fully contained by the different activities, is arguably more accurate, since every participant was walking, running, and cycling at an approximately constant pace. One or two full minutes of step data should be representative of the whole activity period.

A previous study by Wagner et al. demonstrated that the virtual pedometer in two different Android phones, when carried in a waist pouch, was valid for walking speeds above 3 km/h [22]. However, this study did not evaluate pedometer performance across different phone placements. The present study demonstrated that the steps registered by the pedometer were non-significantly different across all phone placements when walking normally, walking briskly, or running (Figure 10). Notably, the phone placements of backpack and fanny pack remained non-significantly different from the SENS Motion^®^ activity tracker across all activities. Moreover, using the fully contained step method, the step count was more comparable to the SENS Motion^®^ step count, as evident in Figure 9 compared to Figure 10.

### 4.5. Limitations

Participants were recruited from the Department of Electrical and Computer Engineering at Aarhus University and, therefore, consisted primarily of students and faculty members. As a result, the sample was predominantly male and largely within the age range of 20–27 years.

While this population is adequate for a preliminary validation of smartphone placement effects, the sample size and low diversity limit the generalizability of the findings. Inter-individual variability, such as differences in movement patterns, gait characteristics, or sex-related variation in posture, could not be examined. In addition, the sample size did not allow for meaningful analyses of anthropometric factors, and potential associations between height or leg length and SENS Motion^®^ misclassification could therefore not be investigated. Additionally, the participants were healthy individuals without mobility impairments, further limiting the applicability of the results to populations with altered or pathological gait patterns. Overall, these limitations underscore the need for future research to include larger and more diverse samples, including older adults and individuals with disabilities, to improve generalizability.

Participants wore their own clothing to better reflect real-world use. While this strengthens the practical relevance of the findings, it also introduces variability, as pocket fit may differ substantially between individuals due to variations in trouser design and body shape. Such differences may influence the stability of the smartphone within the pocket and thereby affect activity recognition classification. Although this variability mirrors typical real-life conditions and would also be present in larger field-based studies, the inability to standardize pocket tightness remains a methodological limitation and should be considered when interpreting placement-dependent results. The experiment was conducted in a controlled laboratory setting, and step counts were derived from treadmill-based walking and running. Step counts observed during real-world activities on varying floor types and surfaces may therefore differ from those obtained under laboratory conditions [22]. In addition, smartphone step counts were stored on a per-minute basis, which constitutes a technical limitation in the context of this study. This required the use of interpolation methods to estimate steps within specific activities, introducing a systematic “smearing” of data across activity transitions. This explains the observed underestimation of steps during active periods and the corresponding overestimation during stationary periods. Consequently, step count agreement is likely impacted by this methodological constraint and should therefore be interpreted with caution in these cases.

Extending the duration of each activity would likely improve the statistical robustness of the results and reduce the relative impact of inherent transition latencies associated with the smartphone APIs. This latency is particularly evident in the transition to “Still” activity, where the devices may require more time to confirm a state of inactivity than the protocol of this study provided. Furthermore, evaluating a wider range of running and cycling speeds may be necessary, as the selected running speed (8 km/h) may have been insufficiently distinct from walking. While this speed was chosen to ensure that all participants could complete the activity comfortably in their regular clothing, it may have been slower than participants’ natural running pace, which may have caused some people to run unnaturally, further impacting the results. Nevertheless, the scope of the experiment was deliberately limited to facilitate recruitment and ensure adequate sample size.

This study does not fully simulate natural, everyday movement patterns, such as outdoor running or cycling. This is an important limitation, as outdoor movement and movement on different surface types can differ from controlled laboratory conditions. Future research should investigate outdoor activities in real-world environments to further validate the systems. This limitation is particularly relevant for the cycling task, performed on a stationary bike, which does not include the initial forward acceleration present during outdoor cycling. This may influence activity recognition performance for both the smartphone-based system and the SENS Motion^®^ activity tracker. Finally, the BeSAFE+ app has been developed for Android devices but also needs to be made available for iOS devices, as the majority of the population, e.g., in Denmark, uses an iOS phone (Mobile Operating System Market Share Denmark: Mobile Operating System Market Share Denmark | Statcounter Global Stats (https://gs.statcounter.com/os-market-share/mobile/denmark#monthly-202001-202501, accessed on 10 April 2025)). Since the app is already developed using the cross-platform framework, MAUI, it could be extended to iOS devices with relative ease. The BeSAFE+ app could also be extended to support smartwatches. However, the performance of the Sampling API and Transition API must be evaluated when using a smartwatch for HAR.

## 5. Conclusions

Overall, the SENS Motion^®^ activity tracker demonstrated higher classification accuracy than most smartphone placements across all activities, except for running. Nevertheless, several smartphone placements and API approaches achieved activity recognition and step count estimates that were not significantly different from the SENS Motion^®^ activity tracker. During stationary cycling, placing the smartphone in the front pocket combined with the Sampling API achieved the highest classification accuracy and showed activity classification comparable to the SENS Motion^®^ activity tracker. Using the Transition API with delay-compensation, the backpack and fanny pack outperformed the SENS Motion^®^ activity tracker during running and achieved comparable results for walking (normal and brisk). Furthermore, step counts derived from the fully contained steps approach for the backpack and fanny pack placements did not differ significantly from those obtained with the SENS Motion^®^ activity tracker across all activities. As hypothesized, the classification accuracy varied across the different phone placements, however the armband did not achieve a lower classification accuracy. Instead, it showed a non-significant difference from the SENS Motion^®^ activity tracker during walking using the Sampling API with LOCF, and running using all three analyzed APIs.

### Implications

These findings indicate that smartphone-based monitoring may serve as a practical alternative in situations where dedicated thigh-mounted sensors are unavailable or impractical due to cost, battery limitations, the need to replace the adhesive patch, or logistical constraints such as fewer in-person follow-up visits at hospitals or rehabilitation centers. While no single smartphone placement was optimal for all activities, the results provide novel guidance for clinicians and researchers on how to position the device according to the activity of interest. However, it is important to note that the optimal choice of API and placement depends on the specific clinical priority, and the inherent variability in smartphones may affect the generalizability of the delay-compensation parameters. For stationary cycling, the front pocket is recommended; for general walking, the fanny pack is preferable; and for running, the backpack or fanny pack. Further research is warranted in larger and more diverse patient populations, and under real-life conditions, to confirm these findings and extend them to other physical activities relevant to rehabilitation and long-term health monitoring.

## Figures and Tables

**Table 1:** Activity descriptions and invalid phone placements.

Order	Activity	Minimum Duration	Correct Activity Label	Description	Invalid Phone Placement
1	Normal walking	2 min	Walking	Walking on a treadmill with the speed set to 3.5 km/h	N/A
2	Standing still	1 min	Still	Standing still in an upright position	N/A
3	Brisk walking	2 min	Walking	Walking on a treadmill with the speed set to 5.5 km/h	N/A
4	Sitting still	1 min	Still	Sitting down in a chair sideways such that there is space for the backpack	N/A
5	Running	2 min	Running	Running on a treadmill with the speed set to 8 km/h	N/A
6	Lying still	1 min	Still	Lying down in bed	Backpack
7	Cycling (low intensity)	2 min	OnBicycle	Cycling on an exercise bike at a normal and comfortable pace between 40 and 50 RPM	N/A
8	Cycling (high intensity)	2 min	OnBicycle	Cycling on an exercise bike at a fast pace between 80 and 90 RPM	N/A

RPM: revolutions per minute.

**Table 2:** SENS Motion^®^ activity categories divided into Transition API defined activities for comparison.

Transition API Defined Activities	SENS Motion^®^ Activity	Description
Still	activity/lying/time	The patient is lying down.
	activity/sitting/time	The patient is sitting down.
	activity/upright stand/time	The patient is standing.
Walking	activity/upright move/time	The patient is standing with some movements. This activity is between ‘Standing’ and ‘Walking’ but is irregular.
	activity/upright walk/time	The patient is walking continuously.
	activity/upright stair/time	The patient is walking up/downstairs.
Running	activity/upright run/time	The patient is running.
On Bicycle	activity/cycling/time	The patient is cycling.
Unknown	activity/row/time	The patient is rowing.
	activity/non wear/time	The patient is not wearing the sensor.

**Table 3:** Participants characteristics.

Variable	Total *n* = 20
Age, years	26 (20–52)
Sex, male, *n* (%)	17 (85%)
Weight, kg	76 (50–98)
Height, cm	180 (161–195)

Data presented as mean and range unless otherwise stated.

## Data Availability

Data and source code can be provided upon request subject to Aarhus University rules and regulations.

## Abbreviations

The following abbreviations are used in this manuscript:

HAR	Human Activity Recognition
LOCF	Last Observation Carried Forward
PA	Physical activity
RPM	Revolutions per minute
Sampling API	Activity Recognition Sampling Application Programming Interface
Transition API	Activity Recognition Transition Application Programming Interface

## Appendix A

**Table A1:** Patient characteristics.

Participant ID	Age (cm)	Sex	Weight (kg)	Height (cm)
1	27	male	80	187
2	27	female	58	177
3	52	male	75	180
4	20	male	89	185
5 *	21	female	50	161
6	23	male	98	188
7 *	21	male	82	182
8	24	male	86	183
9	21	male	70	169
10	21	male	87	195
11 *	23	male	86	186
12	25	male	70	182
13	24	male	79	184
14	46	male	80	180
15	23	female	62	169
16	24	male	82	191
17	25	male	68	177
18	24	male	64	172
19	27	male	79	173
20	25	male	80	182

* Participants did not have a suitable back pocket and data from that placement was excluded for those participants.

**Table A2:** Kruskal–Wallis *p*-values for different activities.

Activity	Sampling API	Transition API (Raw)	Transition API (Compensated)
All	<0.001	<0.001	<0.001
Still	<0.001	<0.001	<0.001
Walking	<0.001	<0.001	<0.001
Running	<0.001	<0.001	<0.001
On Bicycle	<0.001	<0.001	<0.001

**Table A3:** Kruskal–Wallis *p*-values for different observed activities.

Observed Activity	Sampling API	Transition API (Raw)	Transition API (Compensated)
Lying	<0.001	<0.001	0.019
Sitting	<0.001	<0.001	<0.001
Standing	<0.001	<0.001	<0.001
Walking normal	0.014	<0.001	0.029
Walking brisk	0.016	<0.001	<0.001
Running	<0.001	<0.001	<0.001
On Bicycle Low	<0.001	<0.001	<0.001
On Bicycle High	<0.001	<0.001	<0.001

**Table A4:** Kruskal–Wallis *p*-values for smoothed steps across observed activities.

Observed Activity	*p*-Value
still_standing	<0.001
still_lying	<0.001
still_sitting	<0.001
walking_normal	<0.001
walking_brisk	<0.001
running	<0.001
On_bicycle_slow	<0.001
On_bicycle_high	<0.001
